# The MEK Inhibitor Trametinib Improves Outcomes following Subarachnoid Haemorrhage in Female Rats

**DOI:** 10.3390/ph15121446

**Published:** 2022-11-22

**Authors:** Jesper Peter Bömers, Anne-Sofie Grell, Lars Edvinsson, Sara Ellinor Johansson, Kristian Agmund Haanes

**Affiliations:** 1 Department of Clinical Experimental Research, Glostrup Research Institute, Copenhagen University Hospital—Rigshospitalet, Nordstjernevej 42, DK-2600 Glostrup, Denmark; 2 Department of Neurosurgery, Copenhagen University Hospital—Rigshospitalet, Blegdamsvej 9, DK-2100 Copenhagen, Denmark; 3 Department of Clinical Sciences, Division of Experimental Vascular Research, Lund University, 221 84 Lund, Sweden

**Keywords:** ET-1, cerebral artery, rat, SAH, subarachnoid haemorrhage, trametinib

## Abstract

Aneurysmal subarachnoid haemorrhage (SAH) is a haemorrhagic stroke that causes approximately 5% of all stroke incidents. We have been working on a treatment strategy that targets changes in cerebrovascular contractile receptors, by blocking the MEK/ERK1/2 signalling pathway. Recently, a positive effect of trametinib was found in male rats, but investigations of both sexes in pre-clinical studies are an important necessity. In the current study, a SAH was induced in female rats, by autologous blood-injection into the pre-chiasmatic cistern. This produces a dramatic, transient increase in intracranial pressure (ICP) and an acute and prolonged decrease in cerebral blood flow. Rats were then treated with either vehicle or three doses of 0.5 mg/kg trametinib (specific MEK/ERK1/2 inhibitor) intraperitoneally at 3, 9, and 24 h after the SAH. The outcome was assessed by a panel of tests, including intracranial pressure (ICP), sensorimotor tests, a neurological outcome score, and myography. We observed a significant difference in arterial contractility and a reduction in subacute increases in ICP when the rats were treated with trametinib. The sensory motor and neurological outcomes in trametinib-treated rats were significantly improved, suggesting that the improved outcome in females is similar to that of males treated with trametinib.

## 1. Introduction

Aneurysmal subarachnoid haemorrhage (SAH) is a haemorrhagic stroke that causes approximately 5% of all stroke incidents. Although the total SAH percentage is relatively low, it has a mortality rate of approximately 40%, which often affects younger individuals compared to other stroke types [1]. Among survivors, cognitive impairments have a great impact on the quality of life, and since many of those affected are relatively young, it makes a SAH a significant cause of years lived with disability [2,3]. The overall SAH incidence is higher in women compared to men and, furthermore, the mortality rate in women is also elevated [4], but the outcome following the occurrence of a SAH might not be different [5]. The increased risk in women is suggested to be due to a general increase in the risk of aneurism development due to hormonal changes or intrinsic wall weakness [6]. Despite this, most of the experimental research has focused on males. Moreover, compared to the distribution of strokes in the population, women are underrepresented in trials for the disease [7].

The pathophysiology of a SAH is multifaceted. In the initial phase, usually caused by the bursting of an aneurism, blood leaks out into the subarachnoid space, which leads to acutely increased intracranial pressure (ICP). Although the cerebral blood flow (CBF) is regulated by several mechanisms [8], the increase in pressure following a SAH reduces CBF, leading to ischemic damage [9]. Following a short recovery phase, many patients develop a secondary delayed brain injury, or delayed cerebral ischemia (DCI) [10] associated with inflammation [11], oedema [12,13], blood–-brain barrier (BBB) disruption, and vasospasms associated with an increased expression of contractile receptors/vascular phenotypic changes [14]. Our research group has focused on a delayed brain injury after a SAH and primarily the associated vascular phenotypical changes. Interestingly, angiographic or symptomatic vasospasm is also more prevalent in women [4,15].

The most striking vascular change is the upregulation of vasocontractile endothelin B (ET_B_) and serotonin (5-HT_1B_) receptors [14,16], which are associated with increased vasoconstriction and diminished CBF [17]. Acutely high ICP [18] and high phosphorylated ERK1/2 (pERK1/2) in the cerebrovascular smooth muscle cells [17] are linked to the phenotypic upregulation of vascular receptors. A treatment strategy targeting these changes, by blocking the MEK/ERK signalling pathway, has been shown to prevent the phenotypic receptor alterations and outcomes in experimental models with great success [16]. The initial experiments used the MEK/ERK inhibitor U0126, which was discovered some decades ago [19]. Recent work has focused on the next generation of MEK1/2 inhibitors, such as trametinib (GSK1120212) [20], which is a specific MEK/ERK inhibitor developed for cancer treatment [21] and prevents ERK phosphorylation in vivo [22]. Trametinib, like U0126, showed an improved outcome following SAHs in male rats. However, the scientific community has recently agreed that investigating both sexes in pre-clinical studies is a necessity [23]. To date, the only female data have come from the middle cerebral artery occlusion model, where despite not reducing infarct size, it showed significantly improved neurological function as well as reducing the phenotypic vascular receptor alterations [24,25,26,27]. In the present study, our aim was to evaluate the possibility that intraperitoneal (i.p.) trametinib may alleviate the outcome of an experimental SAH in females and, secondly, to compare our findings with previous data in male rats, elucidating the general possibility of MEK inhibition between the sexes. 

## 2. Results

### 2.1. Organ Culture

**Figure 1:**
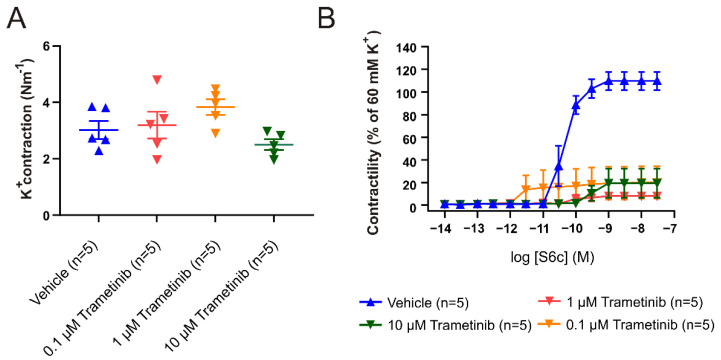
Inhibitory capacity of trametinib after 48 h of organ culture. (**A**) Maximal contraction to 60 mM K^+^ following incubation with 0.1, 1, and 10 µM of trametinib compared to vehicle. There is no significant difference between the vehicle and trametinib. (**B**) Concentration–response curves to the ET_B_ specific agonist S6c, following incubation with 0.1, 1, and 10 µM trametinib. Data are shown as mean ± SEM (*n* = 5).

In several studies, we have shown that the ET_B_ receptor is phenotypically altered to a contractile type and is upregulated following 48 h of flow cessation [14]. The increase in functional contractility following organ culture (OC) is shown by contraction induced by cumulative administration of the specific ET_B_ agonist, sarafotoxin 6c (S6c) (characterized in previous studies, including [20]). The initial experiments aimed to characterize the effect of OC in arteries from female rats (isolated from five rats), combined with testing three different concentrations of trametinib (0.1, 1.0, and 10 µM). There was no significant effect on the contraction induced by 60 mM K^+^ (Figure 1A). The data clearly show that the contractile response to S6c is strong only in the arteries exposed to vehicle. Furthermore, trametinib had a strong inhibitory effect, with a dose of 1.0 µM sufficient to completely prevent the occurrence of contractility in response to the ET_B_ receptor subtype specific agonist sarafotoxin 6c (Figure 1B). Furthermore, the data show that trametinib is easily dissolvable in the cremophor-based vehicle. This demonstrates the shift in contractility, and is similar to what was observed in male cerebral arteries [20].

### 2.2. Experimental SAH 

**Figure 2:**
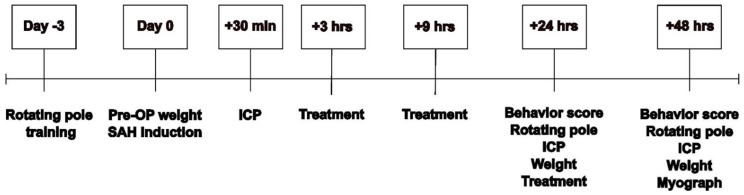
Study outline. Overview of the procedures and analysis performed for the current study. All timepoints relate to time 0, which is the induction of the SAH.

We went further with trametinib in an in vivo treatment study (Figure 2). A total of 44 female rats were included. However, 20 rats were excluded: 6 due to issues related to anaesthesia and surgery, 5 animals did not show a decrease in CBF, 7 did not regain consciousness after surgery, and 2 rats had hemiparesis following surgery. Trametinib was given i.p. at 3, 9, and 24 h following the SAH, with each dose being 0.5 mg/kg. In a rat of 265 g (and approximately 191 mL of body water), this concentration will have an initial distribution of ~ 1.1 µM in the rat. The vehicle was given at the same timepoints and volume. The administration was randomized and blinded to the researchers.

### 2.3. Standard Clinical Parameters

In the 24 animals used for the study (12 SAH + vehicle and 12 SAH + trametinib), the cortical blood flow decreased as a result of the blood injection (% decrease), both in the SAH + vehicle group (91 ± 3%) and the SAH + trametinib group (89 ± 3%; *p* > 0.05). For a typical sample traces and experimental setup, see Bomers et al. [28]. There were no significant differences in physiological parameters measured during surgery between SAH + vehicle and SAH + trametinib: weight (261 ± 6 g vs. 260 ± 6 g), MABP (108 ± 5 mmHg vs. 107 ± 6 mmHg), pH (7.35 ± 0.01 vs. 7.36 ± 0.02), pO_2_ (19.1 ± 1.3 kPa vs. 15.8 ± 0.8 kPa), and pCO_2_ (5.3 ± 0.2 kPa vs. 5.1 ± 0.3 kPa). 

### 2.4. Effect of Trametinib Treatment on Vascular Contractile Responses

**Figure 3:**
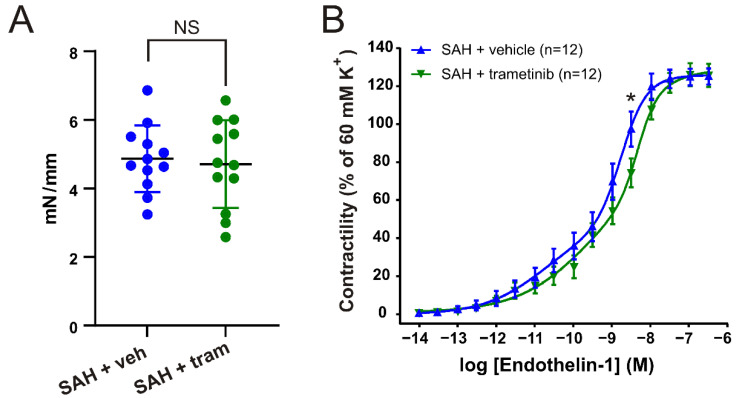
Comparison of trametinib and vehicle treatment on contractility in the BA. (**A**) Maximal contraction to 60 mM K^+^ for the BAs. (**B**) Cumulative concentration–response curves to ET-1 (10^−14^–10^−7^ M) on the BA. Data are shown as mean ± SEM, with statistics calculated using two-way ANOVA followed by Bonferroni post-test, * *p* < 0.05.

There was no significant difference in the 60 mM K^+^-induced contraction per length (Figure 3A) in the BA (SAH_veh_ 4.90 ± 0.23 vs. SAH_tram_ 4.63 ± 0.29; *p* = 0.47). The agonist-induced contractions were therefore normalized to the individual K^+^ response. We compared the cumulative concentration–response curves to ET-1 (10^−14^–10^−7^ M) in BAs from rats treated with either vehicle or trametinib. We observed a significant reduction in the contractility to ET-1 in the trametinib-treated animals (Figure 3B). Combined with a significant shift in the logEC_50_^2^ from −8.77 (95% CI, −8.83 to −8.72) in SAH + vehicle to −8.34 (95% CI, −8.54 to −8.17) in SAH + trametinib, this shows that the threshold for contraction is lower in the response to ET-1 in BAs from trametinib-treated animals. There was no significant change in the logEC_50_^1^, as the CI was large, but the numerical value for SAH + trametinib (−9.53) was higher than for SAH + vehicle (−10.76), indicating a shift.

**Figure 4:**
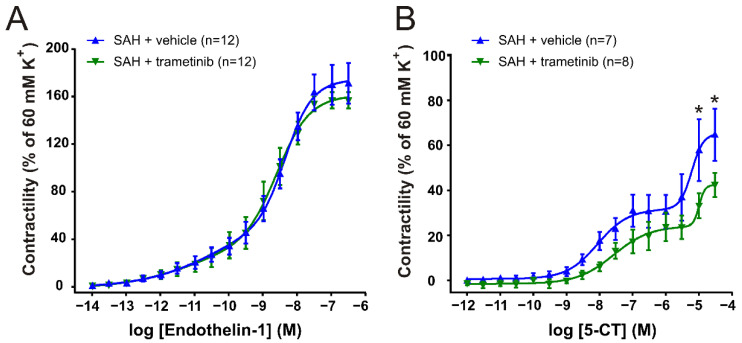
Comparison of trametinib and vehicle treatment on contractility in the MCA (**A**) Cumulative concentration–response curves to ET-1 (10^−14^–10^−7^ M). (**B**) Cumulative concentration–response curves to 5-CT (10^−12^–3 × 10^−5^ M). Data are shown as mean ± SEM, with statistics calculated using two-way ANOVA followed by Bonferroni post-test, * *p* < 0.05.

Although there was a minor numerical reduction in the contractility in the MCA, the K^+^ responses were not significantly different (SAH_veh_ 2.71 ± 0.26 vs. SAH_tram_ 2.09 ± 0.24; *p* = 0.09). For the ET-1 response on the MCAs (Figure 4B), both curves had a preferred fit of the biphasic slope and no significant differences were observed. For cumulative concentration–responses to 5-CT (10^−12^–3 × 10^−5^ M) obtained in MCAs (Figure 4B), there was no significant difference between the treatment groups in logEC_50_^1^ (SAH_veh_ −8.06 vs. SAH_tram_ −7.56) or log EC_50_^2^ (SAH_veh_ −5.22 vs. SAH_tram_ −5.00). However, a significantly higher (*p* = 0.033) maximal contractility was observed in MCAs from rats treated with vehicle (E_max_ 64.5 ± 11.5% of K^+^ contraction) compared to MCAs from the SAH + trametinib group (E_max_ 42.2 ± 5.35% of K^+^ contraction). For the response to 5-CT, we excluded arteries that did not show a response (defined as 20% contraction). There were 5 rats excluded in the SAH + vehicle group and 4 rats in the SAH + treatment group.

### 2.5. Elevated Intracranial Pressure in the Subacute Phase after SAH in Female Rats

**Figure 5:**
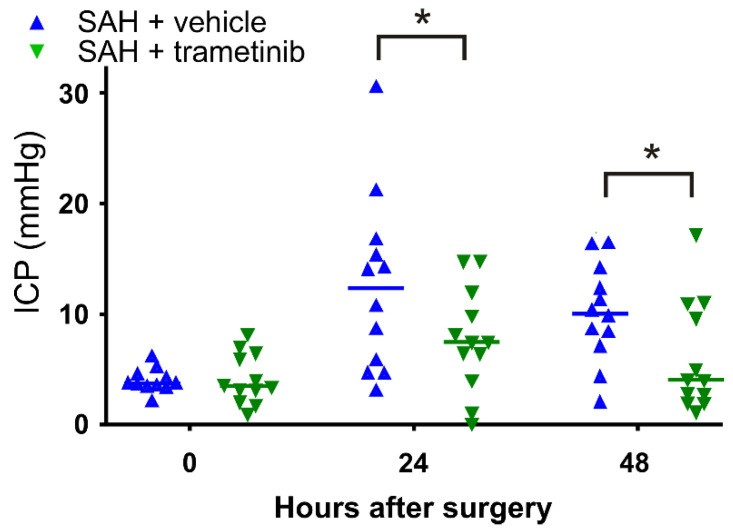
Intracranial pressure post-SAH. The average ICP is significantly higher after 24 h in both groups. The increase in ICP is less in the rats treated with trametinib and, at 48 h, it is only significantly increased in the SAH + vehicle group. A multiple t-test with correction (* *p* < 0.05) was used for statistical analysis.

We measured the intracranial pressure in the rats 30 min post-SAH and at 24 h and 48 h following the SAH. There was no difference in the initial starting point between the SAH + vehicle (4.0 ± 0.3 mmHg) and SAH + trametinib (4.2 ± 0.7 mmHg) group (Figure 5). The ICP increased 24 h after the SAH in both the SAH + vehicle and SAH + trametinib groups (to 12.5 ± 2.3 mmHg and 7.7 ± 1.4 mmHg, respectively). We observed a tendency toward normalization 48 h after the SAH in the SAH + trametinib group (return to 6.1 ± 1.4 mmHg), and there was no longer a significantly increased ICP in these animals compared to the starting point in this group (*p* = 0.50). In contrast, only a minor recovery (to 10.1 ± 1.3 mmHg) was observed in the SAH + vehicle group, and there was still a significantly increased ICP (*p* = 0.001) compared to the starting point. The difference was significant 48 h after the SAH (*p* < 0.05) due to the greater reduction in ICP for the SAH + trametinib group. 

### 2.6. Weight Loss and General Well-Being

**Figure 6:**
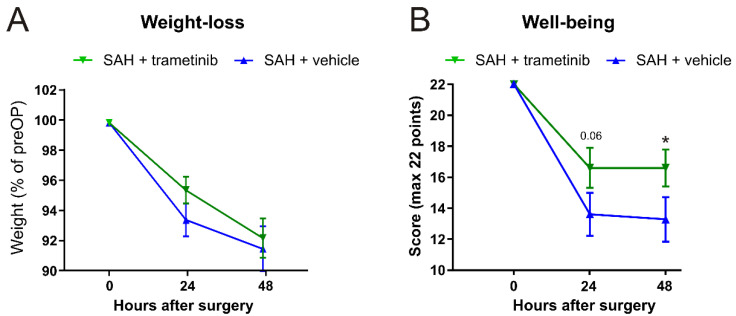
Weight loss and general well-being. (**A**) Animals were weighed at the starting point and after 24/48 h. There was a significant weight loss in both groups, with no significant effect of the trametinib treatment. (**B**) The general well-being ratings used a 22-point scoring system. Multiple t-tests with multiple testing correction (* *p* < 0.05) were used for statistical analysis.

Loss of weight could be linked to dysphagia, decreased dietary intake, and other neurologic deficits that contribute to eating difficulties [29], and the animals typically lose weight due to a SAH. We observed weight loss in both the SAH + vehicle (8.5 ± 1.5%) and SAH + trametinib (7.8 ± 1.3%) groups after 48 h (Figure 6A). The animals were also subjected to a further test of general well-being and the rats were scored before surgery (time 0) and at 24 h and 48 h after the SAH (Figure 6B). We observed a significant decrease in well-being from 24 h after the SAH in both groups. Despite a higher numerical score for the animals treated with trametinib after 24 h, there was no significant difference (*p* = 0.06). However, at 48 h after surgery, the treated animals recovered slightly, leading to a significant difference between SAH + vehicle (score 13.3 ± 1.4) and SAH + trametinib (score 16.6 ± 1.2). 

### 2.7. Sensorimotor Cognition after SAH in Female Rats

**Figure 7:**
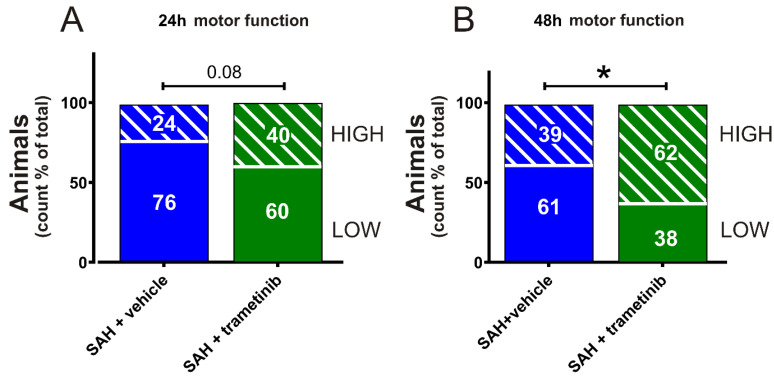
Evaluation of neurologic function post-SAH. Rotating pole test at 10 rpm for: (**A**) 24 h post-SAH and (**B**) 48 h post-SAH. Scored with 4 counts per animal, i.e., 2 scores for left and right rotation, respectively; Low = Unable to traverse in one try; High = Able to traverse in one try. Fisher’s exact test (* *p* < 0.05) was used for statistics.

We further evaluated neurological deficits by using the rotating pole test. The rats were scored 24 h (Figure 7A) and 48 h (Figure 7B) after the SAH (data are shown as % high score count/total score count). At the 48 h endpoint, the SAH + vehicle group (High score_48h_ 39%) had a significantly lower score (*p* = 0.027) compared to the SAH + trametinib group (High score_48h_ 62%). The rotating pole test is not a pure motor function test, as it requires training of the rats in advance, and therefore requires memory to succeed [30]. When combined, the results suggest that memory, motivation, and sensory motor function are improved when rats are treated with trametinib compared to vehicle. 

## 3. Discussion

This is the first study to investigate the effect of a highly specific MEK/ERK1/2 inhibitor, trametinib, in female rats subjected to experimental SAH. We show that there is an overall improved outcome when treating with trametinib, administered 3, 9, and 24 h after SAH induction. 

### 3.1. Contribution to the Field

In light of the large female population in SAHs that is highly understudied and undertreated, we show here that this new paradigm results in outcomes similar to male rats and that the acting mechanism is equally active in either sex. In previous studies, we compared a MEK1/2 inhibitor with the commonly used dihydropyridine type of calcium channel inhibitor, nimodipine [31], and compared it to a specific endothelin receptor blocker, clazosentan [32]. The outcome was in favour of the MEK/ERK1/2 inhibitor versus the other two agents. Thus, combined with the current work, this has led to an opening for an approach alleviating the consequences of SAHs in both males and females.

We have long postulated that upregulation of contractile receptors following a SAH is an important contributing factor to the development of DCI and that inhibition of the MEK/ERK pathway may prevent the subsequent increased vasoconstriction and thereby also prevent DCI [14,33]. These mechanisms are not unique to larger cerebral arteries, as a similar increase in ET_B_ receptors is also observed in the intraparenchymal vasculature [34]. Although acute SAH mortality is the most common cause of death, DCI is a major cause of secondary mortality and poor outcomes [9]. In concert, we observed vascular differences between the SAH + vehicle and SAH + trametinib groups. There was a significant reduction in the contractile responses to ET-1 in the BA following trametinib treatment, and a reduction in the response to 5-CT in the MCA. Compared to Spray et al. [35], the current study demonstrated a lower contractile effect of ET-1 and 5-CT vasoconstriction in SAH + vehicle compared to SAH without treatment. One possible reason for this effect of vehicle could be a positive effect of the vehicle *per se*, cremophor [36,37], and could explain why the differences between the treatment and trametinib are not as large as we have seen with U0126 in DMSO. We have earlier shown that cremophor by itself has an inhibitory effect on the upregulation of vasocontractile receptors ex vivo [38]. This is believed to be caused by an additional effect on PKC [39], which has been shown to be a secondary pathway activated in strokes [40] and SAHs [41].

Furthermore, it is important to keep in mind that, although the changes in contractility might appear minor, the ligands for these receptors have been reported to increase following a SAH. ET-1 and 5-HT levels in CSF in humans following a SAH were higher regardless of sex [42,43]. Targeting, for example, the endothelin A (ET_A_) and B (ET_B_) receptors with the antagonist clazosentan, nevertheless, did not lead to successful therapy [44]. We believe that this is because a broader target is needed; several phenotypical modulations occur after a SAH, so inhibiting the MEK/ERK pathway, which prevents the initiation of these changes, is the right approach [32].

In addition to the DCI, prolonged increase in the ICP has been shown to be an important contributor to worsening complications following a SAH, with one major reason being reduced perfusion pressure in the arteries [45]. ICP above 20 mmHg, in particular, is associated with a high mortality rate following a SAH [46]. This is the reason for the inclusion of ICP as an outcome measure. Indeed, there were two animals in the SAH + vehicle group where clinical intervention would likely have been applied with the aim to reduce the ICP, whereas there were none in the SAH + trametinib group (Figure 5). Aside from a significant reduction in ICP in the SAH + trametinib group, our data show that MEK/ERK inhibition prevents an increase in ICP after 48 h, as there was no longer a significant difference from time 0, in contrast to the vehicle treatment, which still had a significant increase in ICP after 48 h. It has been postulated that high ICP leads to an increased duration of vasospasms [10]. However, based on the current data, one could also suggest that MEK/ERK inhibition ensures normal flow, leading to a lower ICP. This requires further attention.

There have been previous studies on the comparison of severity of SAHs in male and female rodents, but no treatment study exists on MEK/ERK inhibition involving both sexes. Fredrich et al. showed that males had a worse outcome in an endovascular puncture model of SAHs. Importantly, diameter changes in males were reduced by 25%, whereas in females they were reduced by only 12% [47]. In contrast, a follow-up study on three-month-old rats showed that the intensity of a SAH in males and females was similar [48]. In our model of autologous blood injection, when comparing SAHs, we observe that cellular damage is similar between males and females [35,49]. 

When comparing data for male rats, it appears that targeting MEK/ERK is nearly equally effective in males and females in experimental SAHs. This might be surprising, as there have been multiple studies showing differences in vascular wall MEK/ERK1/2 signalling between males and females. For example, looking at signalling with IL-1β, an important part of the pathology of a SAH [50], a study on rat aortic smooth muscle cells showed that pERK1/2 was significantly higher in males than in females after exposure to IL-1β [51], suggesting that the MEK/ERK pathway is not identical between the sexes. The difference in MEK/ERK signalling between males and females is observed in other areas as well, for example, in memory formation [52]. In relation to vascular changes, we have previously shown that upregulation of endothelin receptors is not equally strong in human female arteries compared to males [53]. Despite receptor upregulation not being as strong in females compared to males following a SAH, our animal model shows that trametinib treatment still significantly improves outcomes in female rats following a SAH.

### 3.2. Clinical Relevance

In a previous study, we performed a full screen of almost 10 MEK/ERK1/2 inhibitors and discovered that trametinib was the most potent, with improved outcomes in male rats [20]. In light of this, combined with our previous study, we ask if trametinib is the right choice for a clinical trial for SAHs? The current study concludes that targeting the MEK/ERK pathway is a potential treatment strategy for SAHs, also in females. Although, studies have shown that P-gp transport of trametinib out of the brain in mice occurs [54], particularly at higher concentrations, there is work providing evidence for trametinib having decent permeability through the BBB. The free fraction of trametinib is indeed similar in the plasma and in the brain [55]. This is important, as parts of the cerebral smooth muscle cells are inside the BBB. The concentration used in the current study is higher than that used in cancer studies in humans, where an oral 2 mg dose of trametinib gave a plasma concentration of ∼0.035 μM [56]. Toxicity studies indicate that the lethal dose of trametinib is more than 200-fold of what is used in the current study in rodents. In rats, single (oral) doses up to 10 mg/kg were used without serious adverse events [57]. Hence, higher concentrations of trametinib could potentially be used in human clinical trials for SAHs. What also speaks in our favour is the short-term treatment compared to cancer patients; our model suggests only blocking the MEK/ERK1/2 pathway early (dosing during the first 24 h only), while in cancer therapy, dosing lasts for many months. 

### 3.3. Limitations

The current SAH model might not fully model all aspects of the pathology of SAHs in humans. The main limitation is that the mechanisms of both EBI and DCI are multifactorial and not limited to an ICP increase or to vasospasms, and the pathophysiology of vasospasms is complex. However, available molecular biology on human autopsy cerebral blood vessel specimens lends strong support to the involvement of phenotypic alterations in human stroke and SAH specimens [58]. Further, we do not show that trametinib directly affects the phosphorylation of ERK. Nevertheless, trametinib is an extremely specific inhibitor and has been shown in other studies to reduce ERK *phosphorylation* in vivo [22]. Thus, the most important finding in the current study is the functional data on improved outcomes following a SAH and its relation to clinical implications. Finally, several other factors are important in the development of DCI, including microvascular injury and microthrombosis, cortical spreading depolarization, brain inflammation, oxidative stress, metalloprotease upregulation, and blood–brain barrier dysfunction [9]. Investigating the effect of MEK/ERK inhibition on these parameters could provide useful pre-clinical data.

## 4. Materials and Methods

### 4.1. Animals

A total of 49 female Sprague–Dawley rats (NTac:SD, Taconic Denmark) were used for the study (5 for organ culture, 44 for the surgical procedure, whereof 20 were excluded; see Section 2.1). The rats were housed at a constant temperature (22 ± 2 °C) and humidity (55 ± 10%) with a daily rhythm of 12 h light/12 h dark, and were fed Altromin (Scanbur, Denmark) and water ad libitum. After the surgical procedure, rats were single-housed (Type III with 123-Lid) in Eurostandard cages (Type III with 123-Lid) with 2–6 rats per cage. Before the experiment, all rats were acclimatized for at least two weeks.

### 4.2. Preparation of Trametinib Solution

PEG4000 (8074850050, Merck) and cremophor (238470-1SET, Merck) were heated in a warm bath at +37 °C until fluidity increased, before both were added to NaCl 9 mg/mL in a ratio of 1:1:8. The mixture was sonicated (3 × 15 min). Trametinib (synonyms: GSK1120212, JTP-74057, Mekinist) from Selleckchem (catalog No. S2673, USA) was then added to this mixture and vortexed, creating a 10 mM solution. The mixture with trametinib was reheated to 37 °C and sonicated until dissolved (3 × 15 min). 

### 4.3. Artery Harvest and Organ Culture

The rat basilar artery (BA) was used to determine the concentration–response relationship ex vivo and to confirm the effective concentration of trametinib. Trametinib was dissolved in DMSO to a stock concentration of 10 mM. This formulation of trametinib was further diluted with DMEM (Dulbecco’s Modified Eagle’s Media) to obtain final concentrations of 0.1, 1, and 10 µM. BAs were carefully dissected from rat brains and cut into four equal sized segments (~1.2 mm long). The segments were incubated for 48 h in DMEM with trametinib or vehicle, and the media was changed after 24 h. After 48 h of incubation, the segments were used for ex vivo pharmacology studies. 

### 4.4. Vaginal Smears—Estrous Cycle Determination

On the day of surgery, the estrous cycle was observed by collecting vaginal smears for microscopic analysis of the cell types present. In order to minimize potential experimental variability resulting from variations in oestrogen levels, proestrus female rats were omitted from the investigation. During this phase, oestrogen levels are elevated relative to previous phases of the cycle [59].

### 4.5. Experimental Model of SAH

A SAH was induced as previously described for male rats [49,60], with all details provided in Bomers et al. [28]. Female Sprague–Dawley rats (230–300 g, 14–17 weeks) were anaesthetised by intraperitoneal administration of a ketamine/xylazine mixture (1.5 mL/kg of a 3:2 mixture of Ketamine (MSD Animal Health) (100 mg/mL) and Xylazine (KVP Pharma, Germany)) and then intubated and ventilated with 30% O_2_ and 70% atmospheric air. Blood gas values (PaO_2_, PaCO_2_, and pH) were routinely analysed in a blood gas analyser (ABL80 FLEX, Radiometer, Denmark). Using a controlled heating pad, body temperature was maintained at 37 °C ± 0.5 °C (TC-1000, CWE, Inc., PA, USA). MABP (mean arterial blood pressure) and ICP (intracranial pressure) were continually measured using catheters placed into the tail artery and cisterna magna, respectively, and coupled to pressure transducers, a Powerlab unit, and LabChart software (both from AD Instruments, Oxford, UK). A laser-Doppler blood flow meter probe (Oxford Optronix, UK) was put in a partially drilled hole, anterior to the bregma and 3 mm to the right of the midline, on the skull plate (regularly chilled by saline irrigation during the procedure). Through a second hole drilled 6.5 mm anterior to the bregma in the midline, a 25G Spinocan^®^ cannula (B. Braun Melsungen AG, Germany) was descended stereotactically at a 30° angle to the vertical plane towards a final location immediately anterior to the chiasma opticum. After a 10 min equilibration period, 300 µL of blood was manually removed from the tail catheter and administered into the cannula. The pressure and rate of the blood injections were manually adjusted to raise ICP over the mean of the MABP (approximately 200 mmHg) and generate an acute and protracted decrease in CBF. The rats were then kept under anaesthesia for an additional 30 min. At the conclusion of surgery, the tip of the ICP catheter was sealed with a PinPort (PNP3F22, Instech, US) for future ICP readings, and the tail catheter, cannula, and laser-Doppler probe were removed and incisions were closed. The rats were then revitalized and extubated. Rats received subcutaneous injections of Carprofen (5 mg/kg, Scan Vet, Denmark) and 2.5 mL isotonic saline following surgery and daily thereafter. No cannula was inserted into the pre-chiasmatic cistern, and therefore no blood was injected into sham-operated rats. Rats were kept in individual cages until their decapitation 48 h after surgery.

### 4.6. Neurological Tests

#### 4.6.1. Rotating Pole Test

Gross sensorimotor function was assessed using the rotating pole test as previously described [31]. In brief, movement across a 10 rpm revolving pole (45 mm diameter, 150 cm length) was examined with rats given motivation in the form of a cage containing bedding material from their home-cage (“smells like home”) at the end of the pole. Rat performance was evaluated using the following criteria: Low, the animal cannot traverse the pole without falling off; High, the animal can traverse the pole without falling off. Before surgery, the animals were trained to traverse the pole with ease. At 24 h and 48 h after surgery, each animal was scored twice for left and right rotation, for a total of four counts per animal. Animals were evaluated by staff who were blind to the experimental groups. The data are presented as a percentage (score count/total score count).

#### 4.6.2. Behavioural Observation

Rats were observed at 24 h and 48 h, respectively, and their behaviour was scored according to the following parameters: body temperature, body posture (low or curved back), eyes (closed, dry, or blood), fur (dirty or piloerection), faeces (dry or none), noise (when handling the rat), noise sensitive (hyperactive), temperament (passive, aggressive), movement, balance, and ears (white). All 11 observations were scored as follows: normal state = 0, medium state = 1, and poor state = 2 [61]. 

### 4.7. Harvest of Cerebral Arteries

Rats were decapitated at 48 h under anaesthesia. The brains were promptly extracted and cooled in a cold bicarbonate buffer solution. Brain BAs and middle cerebral arteries (MCAs) were meticulously dissected. The BAs and MCAs were sliced into 1–1.5 mm-long cylindrical pieces and put on a wire myograph for contractility measures [49].

### 4.8. In Vitro Pharmacology

A myograph (Danish Myograph Technology A/S, Denmark) was used to quantify the contractile responses of cerebral arteries by recording the isometric tension in segments of isolated arteries. In a wire myograph arrangement, vessel segments were fixed on two stainless steel wires with a diameter of 40 µm. The segments were then submerged in a bicarbonate buffer solution (37 °C) with the following composition (mmol/L): NaCl 119, NaHCO_3_ 15, KCl 4.6, MgCl_2_ 1.2, NaHPO_4_ 1.2, CaCl_2_ 1.5, and glucose 5.5. The buffer was continuously aerating with 5% CO_2_ in O_2_ to maintain a pH of 7.4. In a three-step method, vessel segments were stretched to the appropriate pretension (2 mN) and then allowed to equilibrate at this tension for approximately 30 min. The vessels were subsequently subjected to a bicarbonate buffer solution containing 60 mM K^+^, which was generated by substituting NaCl for KCl in the previously reported isotonic bicarbonate buffer solution. K^+^-evoked contractile responses served as reference values for normalization of agonist-induced responses and evaluation of the depolarization-induced contractile capacity of vasculature. Only BAs and MCAs with K^+^-induced responses greater than 2 mN and 0.8 mN, respectively, were utilized for further analysis. Precontraction with 5-hydroxytryptamine (5-HT) (Sigma-Aldrich, H9523) (3 ×10^−7^ M) and relaxation with carbachol (Sigma-Aldrich, C4382) were used to evaluate the existence of functioning endothelium in artery segments (10^−5^ M). The presence of a relaxing response to the cholinergic receptor agonist carbachol is indicative of a functioning endothelium. The cumulative administration of the natural ligand for endothelin receptors (ETA/ETB), ET-1 (Bachem, 4040254), in the concentration range of 10^−14^ to 10^−7^ M, yielded concentration–response curves. Similarly, concentration–response curves to 5-carboxamidotryptamine (5-CT) (Sigma-Aldrich, C117), a 5-HT_1B_/5-HT_1D_ agonist, were generated by applying 5-CT at concentrations ranging from 10^−12^ to 10^−5^ M.

### 4.9. Intracranial Pressure Measurements

ICP recordings were performed at 24 h and 48 h post-surgery using a novel fluid-filled sealed-off PinPort system we developed for consecutive real-time ICP measurements in rats. The cisterna magna catheter’s sealed PinPort (PNP3F22, Instech, US) was connected to a pressure transducer through fluid-filled tubing and a PinPort injector (PNP-3M, Instech, US). The pressure transducer was linked to a power lab and the LabChart program recorded the ICP (AD Instruments, Oxford, UK). Rats were sedated with 0.5 mL/kg midazolam (2.0 mg/kg) 15 min prior to ICP recording, then the ICP was recorded for 15 min, the PinPort injector was removed, and the rat was returned to the animal facility.

### 4.10. Weight

The weight of the animal was measured prior to and at 24 h and 48 h after surgery. Changes in weight are shown as percental changes of the pre-surgical weight.

### 4.11. Statistics and Data Analysis

The data are shown as mean standard error of the mean (SEM), with *n* being the number of rats. For in vitro pharmacology experiments, contractile responses are represented as a percentage of the maximal contraction generated by 60 mM K+ compared to the baseline value. Emax is the maximal contractile response produced by an agonist, whereas logEC_50_ is the logarithm of the drug concentration that induced 50% of the maximum response. Using a two-way ANOVA with the Bonferroni post-test, statistical differences between concentration–response curves were determined. When comparing two distinct observations/timepoints, a multiple unpaired t-test (with correction for multiple testing) was employed, except for the rotating pole, for which Fisher’s exact test was utilized. For statistical analysis, Graphpad 9 software was employed. Significant *p*-values were defined as *p* < 0.05.

## 5. Conclusions

Trametinib has previously been demonstrated to be the most potent MEK inhibitor to prevent upregulation of contractile receptors in male arteries. In the current study, we observed a significant difference in arterial contractility and a decrease in subacute increases in intracranial pressure when trametinib was also administered to female rats. Moreover, sensory motor and neurological outcomes in female rats treated with trametinib were significantly better than vehicle-treated female rats, indicating that the improved outcome in females is comparable to that of males treated with trametinib. The current data support the idea that we can expect an improved outcome in females treated with trametinib.

## Data Availability

Not applicable.

## Abbreviations

SAH	Subarachnoid haemorrhage
DCI	Delayed cerebral ischemia
ET-1	Endothelin-1
5-CT	5-carboxamidotryptamine
5-HT	5-Hydroxytryptamine
ICP	Intracranial pressure
IP	Intraperitoneal
CBF	Cerebral blood flow
ETA	Endothelin receptor A
ETB	Endothelin receptor B
MABP	Mean arterial blood pressure
MEK	Mitogen-activated protein kinase kinase
PKC	Protein kinase C
BBB	Blood–brain barrier
BA	Basilar artery
MCA	Middle cerebral artery
